# DNA repair in species with extreme lifespan differences

**DOI:** 10.18632/aging.100866

**Published:** 2015-12-30

**Authors:** Sheila L. MacRae, Matthew McKnight Croken, R.B. Calder, Alexander Aliper, Brandon Milholland, Ryan R. White, Alexander Zhavoronkov, Vadim N. Gladyshev, Andrei Seluanov, Vera Gorbunova, Zhengdong D. Zhang, Jan Vijg

**Affiliations:** ^1^ Department of Genetics, Albert Einstein College of Medicine, Bronx, NY 10461, USA; ^2^ InSilico Medicine, Inc., Johns Hopkins University, Baltimore, MD 21218, USA; ^3^ Department of Biology, University of Rochester, Rochester, NY 14627, USA; ^4^ Division of Genetics, Department of Medicine, Brigham and Women’s Hospital, Harvard Medical School, Boston, MA 02115, USA

**Keywords:** genome maintenance, aging, longevity, RNA-seq, transcriptome, naked mole rat, DNA repair

## Abstract

Differences in DNA repair capacity have been hypothesized to underlie the great range of maximum lifespans among mammals. However, measurements of individual DNA repair activities in cells and animals have not substantiated such a relationship because utilization of repair pathways among animals—depending on habitats, anatomical characteristics, and life styles—varies greatly between mammalian species. Recent advances in high-throughput genomics, in combination with increased knowledge of the genetic pathways involved in genome maintenance, now enable a comprehensive comparison of DNA repair transcriptomes in animal species with extreme lifespan differences. Here we compare transcriptomes of liver, an organ with high oxidative metabolism and abundant spontaneous DNA damage, from humans, naked mole rats, and mice, with maximum lifespans of ∼120, 30, and 3 years, respectively, with a focus on genes involved in DNA repair. The results show that the longer-lived species, human and naked mole rat, share higher expression of DNA repair genes, including core genes in several DNA repair pathways. A more systematic approach of signaling pathway analysis indicates statistically significant upregulation of several DNA repair signaling pathways in human and naked mole rat compared with mouse. The results of this present work indicate, for the first time, that DNA repair is upregulated in a major metabolic organ in long-lived humans and naked mole rats compared with short-lived mice. These results strongly suggest that DNA repair can be considered a genuine longevity assurance system.

## INTRODUCTION

Maintaining the integrity of the genome is among the most critical functions of a cell. The accumulation of DNA damage and mutations in multicellular organisms increases the risk of cancer and is linked to aging [1–3]. Essential genome maintenance functions include cell cycle control, regulation of cellular death and senescence, and DNA damage signaling and repair. DNA repair pathways are highly conserved, and because of their critical importance for cell survival and genome integrity they have long been hypothesized to represent primary longevity assurance systems. Indeed, ample research has been devoted to measuring DNA repair activities in cells and animals from species with differences in maximum lifespan. Most notably, nucleotide excision repair activity in cultured fibroblasts has been reported as strongly correlated with maximum lifespan [4]. However, later, this correlation was mainly attributed to differences between humans and rodents in the utilization of excision repair for removal of UV-induced lesions, which rodents, due to their fur and nocturnal habits, experience at a much lower rate than humans [5]. While many other attempts have been made to demonstrate correlations between DNA repair activities and lifespan, thus far, there has been no conclusive evidence that long-lived humans have DNA repair activities that are superior to those of short-lived mice.

It is now well documented that even among phylogenetically related rodents, extreme differences in lifespan are common. Most notably, the naked mole rat (NMR), a rodent species with an extreme resistance to cancer, has a maximum lifespan of approximately 30 years, i.e., about 10 times the lifespan of mice [6]. In previous work we showed that the gene mutation rate is significantly higher in the mouse than in NMR or human [7]. Because of the extreme longevity and cancer resistance of the naked mole rat, we speculated that these characteristics were based, at least in part, on more effective genome maintenance, which could be mediated by higher expression levels of genes involved in DNA repair. Most DNA repair genes are constitutively expressed [8] at a level that, in human cancer cell lines, was found to be inversely correlated with chromosomal instability [9]. Hence, we considered it of interest to compare the expression of DNA repair genes in vivo between humans and mice as well as between NMR and mice, reasoning that if longevity is controlled mainly by genome maintenance we should find this reflected in the level of DNA repair-related transcripts in vivo.

In this study, we looked for longevity-associated enrichment of genes involved in DNA repair in RNA-seq data sets obtained from liver in the three different species: human, NMR, and mouse. Liver is the target organ of a large number of DNA damaging agents, and chronic liver diseases are characterized by increased oxidative stress [10]. It therefore requires proficient DNA repair processes for its long-term integrity. Our results show that the expression of genes encoding core DNA repair enzymes is indeed significantly higher in human and NMR than in the mouse, and that most DNA repair signaling pathways are upregulated in the long-lived species. This work provides further evidence that DNA repair functions as a longevity assurance mechanism in mammals.

## RESULTS

**Figure 1:**
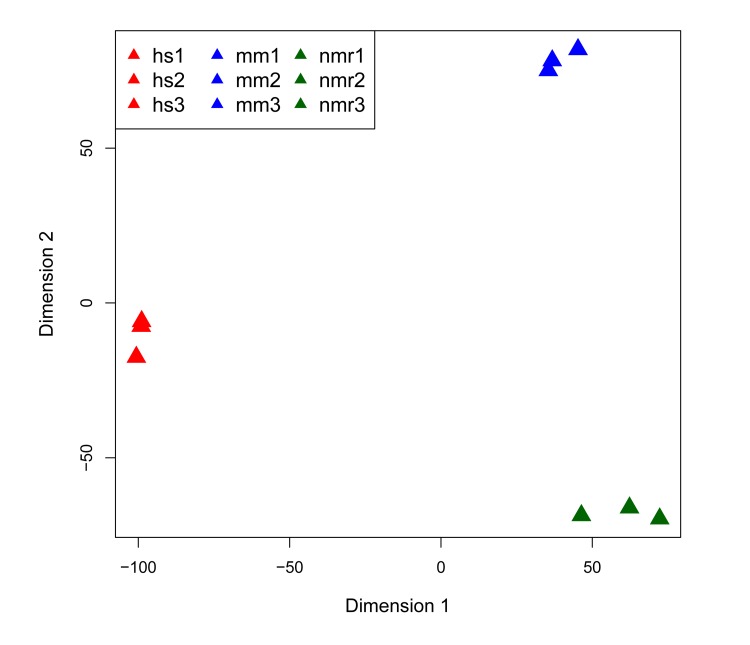
Multi-dimensional scaling (MDS) plot of RNA-seq datasets MDS plot of the 500 highest expressed genes shows that each of the three species clusters together in first two dimensions.

To compare DNA repair gene expression in liver from the three species—human, NMR, and mouse—we produced RNA-seq data for three individuals per species. Figure 1 shows a multidimensional scaling (MDS) plot of the nine sequencing datasets, in which the human, mouse, and NMR samples cluster by species. Table S1 provides details on library sequencing read counts and alignments. Comparing RNA-seq data sets between species poses many challenges. It is particularly difficult to identify orthologs, especially for species with less well-annotated genomes. For our analysis, we used mouse protein-coding genes to identify orthologs in the NMR genome. To identify regions of the NMR genome (hetGla2 [11]) homologous to mouse genes, we isolated mouse entries from UCSC cross-species BLAT results. This provided the genomic coordinates for putative NMR orthologous genes. Initially, there were 16,708 protein-coding mouse genes that mapped to the NMR genome. Of these, 13,993 mouse genes aligned to the NMR genome without overlapping any other alignment. From this list, 13,209 genes exist as putative orthologous gene pairs between mouse and human. We further narrowed this list down to 13,181 genes with unambiguous annotation (Table 1).

**Table 1:** Orthologs compared and their expression in each species

Species	Genes compared	Genes expressed	% expressed
Human	13181	5510	41.80%
Mouse	13181	5459	41.42%
NMR	13181	5496	41.70%

The total number of protein-coding orthologs genes analyzed and the number expressed in each species.

In addition, there are caveats to directly comparing expression of orthologs between species, as biologically the systems are not the same. For example, orthologs may have different gene lengths in the different species, confounding the normalization of read counts. There are also many biological characteristics, such as transcription factors and other gene transcription regulatory mechanisms, that may differ among species and therefore one must be careful in drawing conclusions from direct comparisons of ortholog expression between different species. To mitigate technical biases we used quantile normalization of our read counts, which also factored in gene length [12]. To overcome biological biases that could possibly affect our analysis, we took several approaches to compare expression of DNA repair genes from these three species, including both differential expression analysis and pathway analysis—the latter of which considers all of the genes in a pathway and is less sensitive to biases introduced when directly comparing expression of individual orthologs between species. First, we looked at differential expression of 130 genes encoding proteins characterized as directly involved in DNA repair in mouse and human. Next, we performed a signaling pathway activation analysis with a focus on signaling pathways involved in DNA repair. Finally, to take into account the overall picture of gene expression differences between the three species in liver, we performed an unbiased differential expression analysis of all 13,181 identified protein-coding homologs.

### DNA repair genes are more highly expressed in human and NMR than mouse

**Figure 2:**
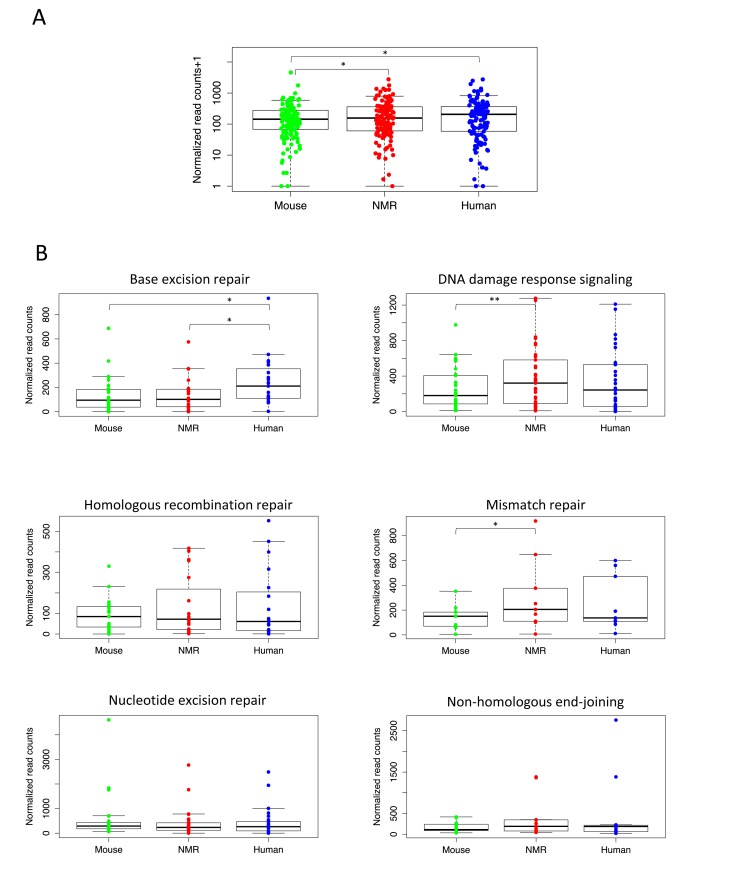
Higher expression of essential DNA repair genes in long-lived species (**A**) Expression of DNA repair genes is significantly higher in livers of naked mole rat (p= 0.0167) and human (p=0.0429) than mouse (Wilcoxon matched-pairs signed rank test). (**B**) For individual DNA repair pathways, human and NMR had higher expression of many essential repair proteins in liver. Human had significantly higher expression than mouse (p= 0.0010) and NMR (p=0.0039) of genes in the BER pathway (Wilcoxon matched-pairs signed rank test), and NMR had significantly higher expression than mouse (p= 0.0496) of genes involved in MMR (paired t test) and DDRS (p=0.0011, Wilcoxon matched-pairs signed rank test).

To determine whether genes involved in DNA repair are more highly expressed in the two long-lived species, we first compiled a set of 130 genes involved in DNA repair activities in mouse and human (Table S2), [13, 14]. In comparing the expression of all of these DNA repair genes among the three species, we found that, as a whole, they are significantly more highly expressed in the longer-lived species, human and NMR, than in the mouse (Figure 2A). By filtering these genes for those with more than two-fold difference in expression, we found that 33 of these genes had significantly higher expression in human than mouse, and 34 had significantly higher expression in NMR than mouse (Tables S4 and S5). Table 2 lists the 12 DNA repair genes having higher expression in both human and NMR than mouse. These include essential enzymes for mismatch repair, base excision repair, non-homologous end-joining, as well as the tumor suppressor gene TP53, which plays an essential role in regulating excision repair pathways [15]. This list also includes five of the eleven human DNA glycosylases, which recognize and remove damaged or incorrect bases [16]. In this analysis mouse had higher expression of only six genes involved in DNA repair (Table S3).

**Table 2:** Essential DNA repair genes having higher expression in human and NMR compared with mouse

Gene symbol	Full gene name
MBD4	methyl-CpG binding domain protein 4
MSH3	mutS homolog 3 (E. coli)
MUTYH	mutY homolog (E. coli)
NEIL1	nei endonuclease VIII-like 1 (E. coli)
NEIL2	nei like 2 (E. coli)
NHEJ1	nonhomologous end-joining factor 1
POLK	polymerase (DNA directed) kappa
POLL	polymerase (DNA directed), lambda
TDG	thymine-DNA glycosylase
TP53	tumor protein p53
UBE2N	ubiquitin-conjugating enzyme E2N (UBC13 homolog, yeast)
XRCC6	X-ray repair complementing defective repair in Chinese hamster cells 6; 70 kDa subunit, Ku70

Includes genes with probability of differential expression greater than 0.95 and fold change greater than two.

We then compared expression of the genes encoding proteins involved in each of the five major DNA repair pathways: base excision repair (BER), mismatch repair (MMR), nucleotide excision repair (NER), homologous recombination repair (HRR), and non-homologous end-joining (NHEJ), as well as DNA damage response signaling (DDRS), which includes genes involved in DNA damage sensing and signaling. Figure 2B shows expression levels of these DNA repair pathways in each species. Our results show that human liver had higher overall expression of genes encoding proteins involved in the BER, HRR, NHEJ, and MMR pathways; however, only the BER pathway expression was significantly higher (p=0.0010). In fact, human liver had significantly higher expression of genes involved in BER than both mouse and NMR. We also found higher expression of HRR and NHEJ genes in NMR than mouse, and genes in the DDRS and MMR pathways show significantly higher expression levels in the NMR than in the mouse (p=0.0496 and 0.0011). This is not surprising, given the extreme cancer resistance of the NMR, and the high rates of cancer in mice. None of the DNA repair pathways had significantly higher expression in mouse than either human or NMR.

### Pathway activation analysis reveals upregulated DNA repair pathways in longer lived species

To test whether signaling pathways involved in DNA repair are upregulated in the long-lived species, we performed a signaling pathway analysis (SPA). This analysis enabled us to compare the differential activation of signaling pathways using a recently published algorithm, OncoFinder, for calculating pathway activation strength (PAS) values [17]. This approach is more biologically informative than comparing overall expression of genes or functional enrichment of differentially expressed genes, as it deals with the functional annotation of each gene product in a pathway and considers its role as an activator or repressor of the signal transduction within the pathway. The absolute value of the PAS indicates the degree of functional changes in the regulation of a signaling pathway, and the sign of PAS indicates whether it is up- or down-regulated, enabling the quantification of signaling changes within a tissue.

**Figure 3:**
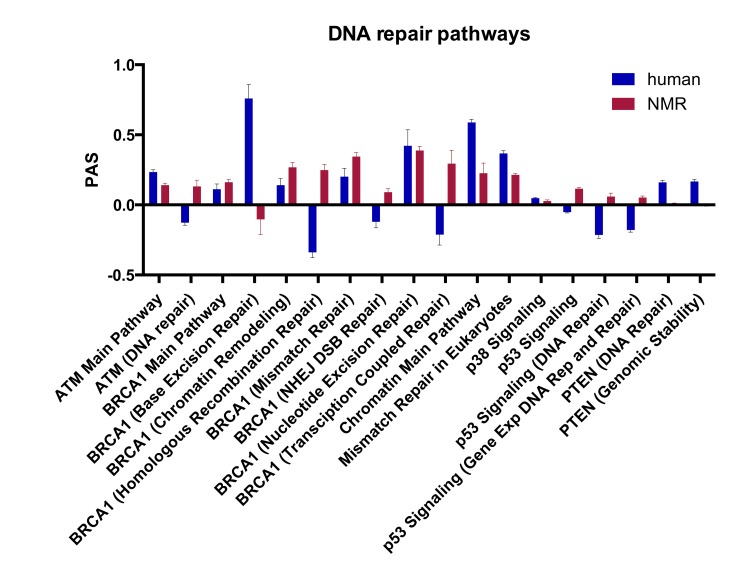
Pathway activation strength (PAS) for DNA repair signaling pathways PAS values for DNA repair pathways in human vs. mouse and NMR vs. mouse (p < 0.05). Positive PAS values reflect upregulated signaling pathways in human or NMR compared with mouse, and negative values represent down-regulated pathways. PAS scores of zero indicate similarly acting pathways in mouse compared with human or NMR.

Using the OncoFinder algorithm we analyzed 375 signaling pathways, annotated from KEGG and SABiosciences, and obtained PAS profiles for human and NMR using mouse as a reference. Of these pathways, over 200 were significantly upregulated in human and NMR compared with mouse (Tables S6 and S7), and from this list we focused on the 18 pathways directly involved in DNA repair. Figure 3 shows the 18 DNA repair PAS profiles for NMR versus mouse, and human versus mouse. Positive PAS values reflect upregulated signaling pathways, and negative values represent down-regulated pathways. PAS scores of zero indicate similarly acting pathways in mouse compared with human or NMR. Sub-pathways for p53, ATM, and BRAC1 are shown in Figure S1. With this SPA approach, we found that all of the DNA repair pathways were upregulated in NMR compared with mouse, except for the BER pathway. Most of the DNA repair pathways were also upregulated in human compared to mouse; however, homologous recombination repair (HRR), non-homologous end-joining (NHEJ), transcription-coupled repair, and the ATM (DNA repair) and p53 pathways were down-regulated in human compared with mouse. The PTEN pathways for DNA repair and genomic stability, and the chromatin main pathway were also more upregulated in human than both NMR and mouse.

### Differential expression analysis and functional annotation

Apart from DNA repair genes and pathways, human, mouse, and NMR are likely to differ greatly in a variety of liver-specific functions. Some of these differences may also relate to the extreme lifespan differences between these species, but most are probably a consequence of differences in lifestyle, habitat, and metabolism. In order to conduct an unbiased differential expression analysis we applied a nonparametric test using the R package NOIseq to compare expression of 13,181 orthologs between the three species [18]. We limited this analysis to genes with normalized expression to counts of 200 or more and a probability of differential expression of 0.95 or higher, and we found that in each of the three pairwise comparisons (mouse vs. human, mouse vs. NMR, and NMR vs. human), around 4,500 genes were differentially expressed (Table 3).

**Table 3:** Differentially expressed orthologs

Comparison	Up in human	Up in mouse	Up in NMR	Differentially expressed
Human vs. Mouse	2056	2181		4237
Human vs. NMR	2294		2426	4720
Mouse vs. NMR		2184	2332	4516

For differential expression orthologs were limited to a read count of 200 or greater and a probability of differential expression of 0.95 or higher.

To look at biological differences in expression of protein-coding genes among human, mouse, and NMR, with a focus on expression in the long-lived species compared to mouse, we performed functional annotation of these differentially expressed genes using G0rilla [19], comparing human with mouse and NMR with mouse. We found 57 gene ontology (GO) biological process terms were significantly upregulated in NMR compared with mouse, and 92 significantly upregulated in human compared with mouse (Tables S8 and S9). We found 35 GO biological process terms that were significantly higher in mouse than NMR, and 30 that were significantly higher in mouse than human (Tables S10 and S11). As expected from gene expression in liver, the majority of the enriched terms were related to metabolism.

**Figure 4:**
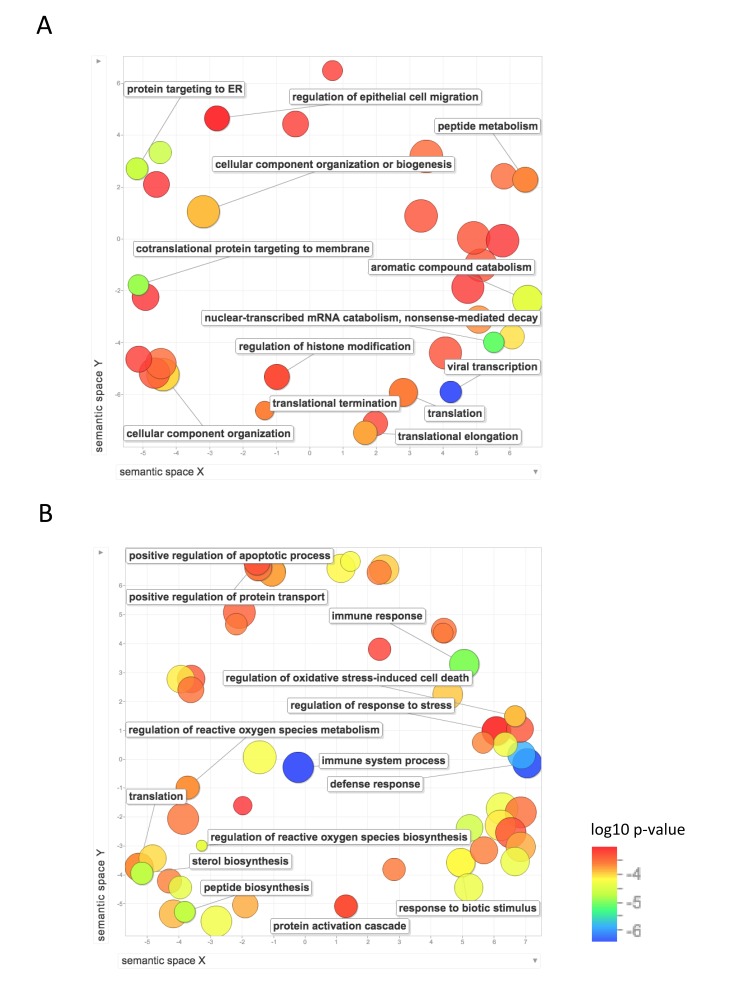
Functional annotation of protein-coding genes more highly expressed in long-lived species Enrichment analysis visualized as an MDS plot for GO biological processes that are (**A**) higher in NMR than mouse (**B**) higher in human than mouse. Plots are generated based on a matrix of semantic similarities in space (x, y). Clusters of circles closer together represent terms that are more closely related. Circle color and size indicates log10 p-value.

We then used REVIGO, a tool that parses GO annotations using semantic similarity, in order to remove redundant terms and to visualize clusters of related terms [20]. Figure 4 shows the GO biological process terms that were significantly upregulated in the long-lived species. Terms that were enriched for higher expression in NMR than mouse include: protein localization and targeting to the endoplasmic reticulum; mRNA and RNA catabolism; translation, including initiation, elongation, and termination; gene expression; and regulation of histone modification and chromatin organization. Terms that were enriched for higher expression in human than mouse include: regulation of reactive oxygen species metabolism, regulation of oxidative stress induced cell death, positive regulation of cell death and apoptotic processes, response to stress, regulation of response to oxidative stress, response to oxygen-containing compound, translation, protein transport, and several biological processes related to immune system response. These results suggest higher regulation of processes involved in gene expression, including chromatin modification, translation, and protein targeting in liver in the long-lived species, and higher response to oxidative stress and regulation of cell death in human liver.

**Figure 5:**
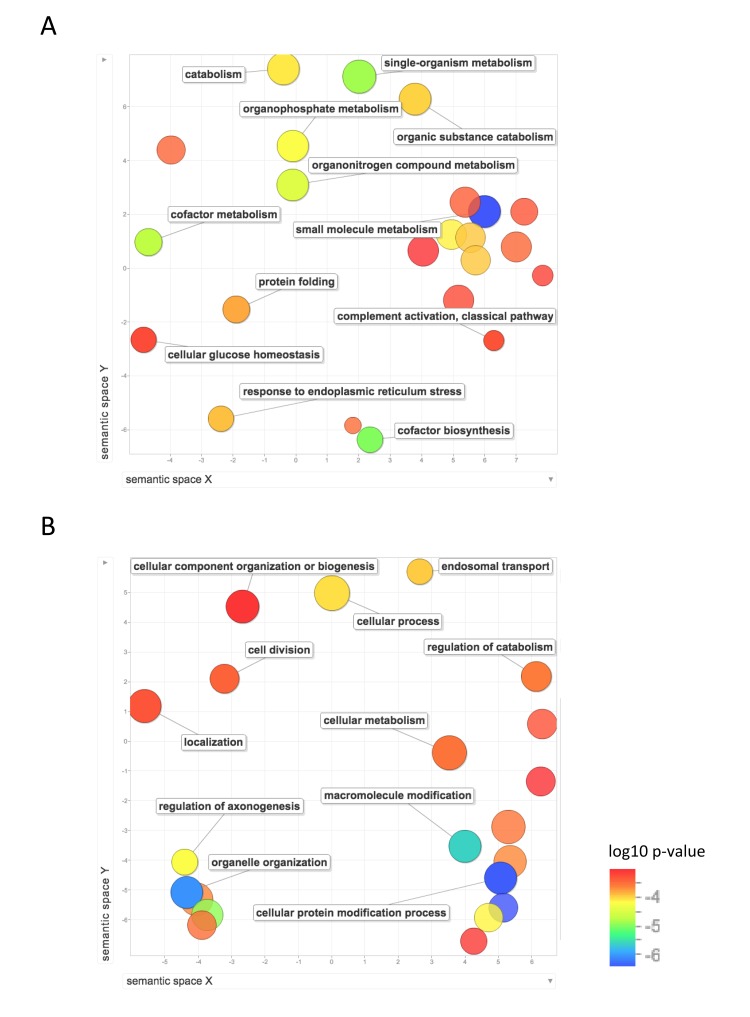
Functional annotation of protein-coding genes more highly expressed in short-lived species Enrichment analysis visualized as an MDS plot for GO biological processes that are (**A**) higher in mouse than NMR (**B**) higher in mouse than human. Plots are generated based on a matrix of semantic similarities in space (x, y). Clusters of circles closer together represent terms that are more closely related. Circle color and size indicates log10 p-value.

Figure 5 shows the GO biological process terms that are significantly upregulated in mouse compared with human and NMR. GO terms enriched for higher expression in mouse than NMR include lipid metabolism, response to endoplasmic reticulum stress, protein folding, oxidation-reduction, cellular glucose homeostasis, and nucleoside metabolism. Compared with human, mouse also showed significant enrichment of GO terms including protein modification, including ubiquitination and de-ubiquitination, chromatin modification, and cell division.

## DISCUSSION

The results of our analysis of the overall expression of DNA repair genes among the three species show that compared with mouse, human and NMR have higher expression levels of DNA repair genes in liver, including significantly higher expression levels of core DNA repair enzymes that are essential for DNA damage sensing and signaling, and the MMR, NHEJ, and BER pathways (Table 2). This supports the hypothesis that the two long-lived species have superior genome maintenance than mouse. The DNA repair genes more highly expressed in the two long-lived species include genes encoding tumor suppressor *TP53*, DNA mismatch repair protein *MSH3*, and NHEJ repair proteins *NHEJ1 and Ku70 (XRCC6)*. We found it interesting that *Ku70* was more highly expressed in human and NMR liver, as *Ku70* mRNA levels have been shown to decline with aging in human cells [21, 22]. This list also includes five of the eleven human DNA glycosylases, *MBD4*, *MUTYH*, *NEIL1*, *NEIL2*, and *TDG*, which are essential for removing and replacing damaged DNA bases. Also more highly expressed in human and NMR than mouse were polymerases λ (*POLL*) and κ (*POLK*). *POLL* is a involved in NHEJ and BER, and is required for cell cycle progression [23], and *POLK* is required for translesion synthesis [24]. We also looked at genes implicated in both longevity and genome maintenance and found that *SIRT6* and *PARP* are more highly expressed in human liver [25].

Most DNA repair genes are not transcriptionally regulated during the DNA damage response, but are constitutively expressed and regulated by post-transcriptional modification. Therefore, having high constitutive levels of mRNA transcripts for these proteins available in the cell is critical for maintaining genome stability. However, some DNA repair genes are transcriptionally induced upon genotoxic stress, including many of the key components of the NER pathway (*DDB1*, *DDB2*, *ERCC1*, *XPC*, *ERCC4*, and *ERCC5*) [26]. This could perhaps explain why we did not find that genes in the NER pathway were more highly expressed in human and NMR compared with mouse. We were also unable to confidently identify two major components of the NER pathway in the NMR genome, *DDB1* and *ERCC1*, so they are unfortunately not included in this analysis. It is important to note that previous studies have shown that rodent fibroblasts have less efficient NER, and specifically global genomic NER (GG-NER), than human cells. However this may not be the case for liver tissue, where NER may be less essential than in UV-exposed skin cells [4, 5].

In our analysis of overall expression of genes in the individual DNA repair pathways, we found that the human and NMR liver samples had higher expression of most of the DNA repair pathways, and that human liver had significantly higher expression of genes involved in BER than both mouse and NMR, which supports our hypothesis that longer-lived species have better DNA repair.

Our findings were confirmed by a more rigorous statistical analysis in which we used the OncoFinder signaling pathway activation algorithm to test whether signaling pathways involved in DNA repair are upregulated in long-lived species compared to the short lived mouse. The results confirm that the long-lived species, human and NMR, have higher activation of DNA repair signaling pathways in liver. The upregulation of nearly all DNA repair pathways in NMR compared with mouse could explain not only its significantly longer lifespan, but also its superior cancer resistance.

We were surprised to find, however, that in our human samples, compared with mouse, the pathways for double-strand DNA break repair, HRR and NHEJ, were down-regulated. In our functional enrichment analysis we found that humans had higher expression of genes involved in GO biological processes regulating cell death and apoptosis, so it is possible that in human liver, DNA double-strand breaks (DSBs) are more likely to lead to cell death than repair. This may be because hepatocytes are predominately quiescent and it is to their advantage to undergo cell death upon suffering DSBs rather than risking cancerous mutations by undergoing error-prone NHEJ repair, the more common DSB repair pathway in mammals used in non-dividing cells. There is evidence that defects in DSB repair contribute to aging in mice as persistent double-strand breaks and chromosomal rearrangements increase in livers of aged mice [27, 28].

In comparing overall gene expression differences of 13,181 orthologous genes through functional annotation, we found that human and NMR have higher expression in liver of genes regulating gene expression, including chromatin modification, RNA decay, translation, and protein localization. It has been shown that NMR has superior translational fidelity than mouse, so it is not surprising to find enrichment for genes regulating translation in the NMR as well [29]. The human liver samples also had higher expression of genes responding to oxidative stress and regulating cell death. As many of these enriched GO terms involve cellular functions related to control of tissue homeostasis and gene expression, and these functions have been shown to deteriorate with age in mice, they possibly reflect the longevity of these species compared with mouse [30]. While the unbiased functional annotation of differentially expressed genes did not show enrichment for DNA repair-related GO biological processes, we speculate that this because in most organs, and especially in liver, the majority of genes expressed are related to cellular metabolism. As these genes show the greatest differences in expression between human and mouse [31], it is difficult to detect other, subtle differences in gene expression for functions such as DNA repair, which involves fewer than 200 genes that are not the most highly expressed. Therefore, functional annotation of differentially expressed genes is perhaps not the best approach for distinguishing expression differences in small sets of genes that are not very highly expressed in either sample.

The results of this study support the hypothesis that the longer-lived species, human and NMR, have superior DNA repair than the short-lived mouse, and confirm that DNA repair is indeed a longevity assurance system.

## METHODS

### Animals and tissue collection

All procedures involving animals were approved by the Institutional Animal Care and Use Committee (IACUC) of Albert Einstein College of Medicine. Three four-month-old Balb/C mice were procured from the National Institutes on Aging. Mice were sacrificed and harvested liver samples were immediately flash-frozen. Three one-year-old naked mole rats were provided by the laboratory of Dr. Vera Gorbunova. They were sacrificed and liver tissues were harvested and immediately flash frozen. Three adult human non-tumor liver samples were obtained from the liver tissue biorepository and Albert Einstein College of Medicine. These samples were preserved in RNA later immediately upon harvesting.

### RNA-sequencing and library construction

Directional RNA sequencing libraries were construction as described previously [28]. Libraries were multiplexed at six samples per lane and sequenced on the Illumina HiSeq.

### Analysis and statistics

To identify regions of the naked mole genome (hetGla2) [11] homologous to mouse genes, we isolated mouse entries from the UCSC cross species BLAT results (http://hgdownload.cse.ucsc.edu/goldenPath/hetGla2/database/xenoRefFlat.txt.gz) [32]. This provided the genomic coordinates for putative NMR homologous genes. We then used UCSC blat search results as NMR "genes". Preliminarily, 16,708 mouse genes (protein coding) mapped to the NMR genome, and 13,993 of these mouse genes aligned to the NMR genome without overlapping any other alignment. From this list, 13,209 genes exist as homologous gene pairs between mouse and human. We narrowed this down to 13,181 genes due to annotation.

Reads were aligned to the NMR (hetGla2), mouse (mm10), and human (hg19) genomes using STAR [33]. Counts were calculated using HTSeq [34]. Read counts were quantile normalized using EDAseq [12], also accounting for gene length within each species.

NOIseq was used to quantify differential gene expression across species [18]. For comparisons of overall expression of genes in specific pathways, the Wilcoxon matched-pairs signed rank test was used for data with non-normal distribution and paired t tests were used when the data were normally distributed.

Gene ontology (GO) analysis was performed using Gorilla, where four genes were required to be considered in each annotation and a *q*-value cutoff < 0.01 [19]. Gene ontology enrichment was visualized using REVIGO with a similarity cutoff set at 0.7 [20].

Pathway activation strength scores were calculated using the OncoFinder algorithm [17], which uses SAbiosciences and KEGG pathway annotations. For this analysis mouse expression data was used as the reference data set.

Figures were generated using R [35] and Prism by GraphPad.

## SUPPLEMENTARY DATA FIGURE AND TABLES
























