# Supplementary material for: DNA repair in species with extreme lifespan differences
Source: Aging (Albany NY). 2015 Dec 30;7(12):1171–82. doi: 10.18632/aging.100866 (PMC4712340; doi:10.18632/aging.100866)
Supplement: Supplementary file 1 [file aging-07-1171-s001.pdf]

SUPPLEMENTARY DATA

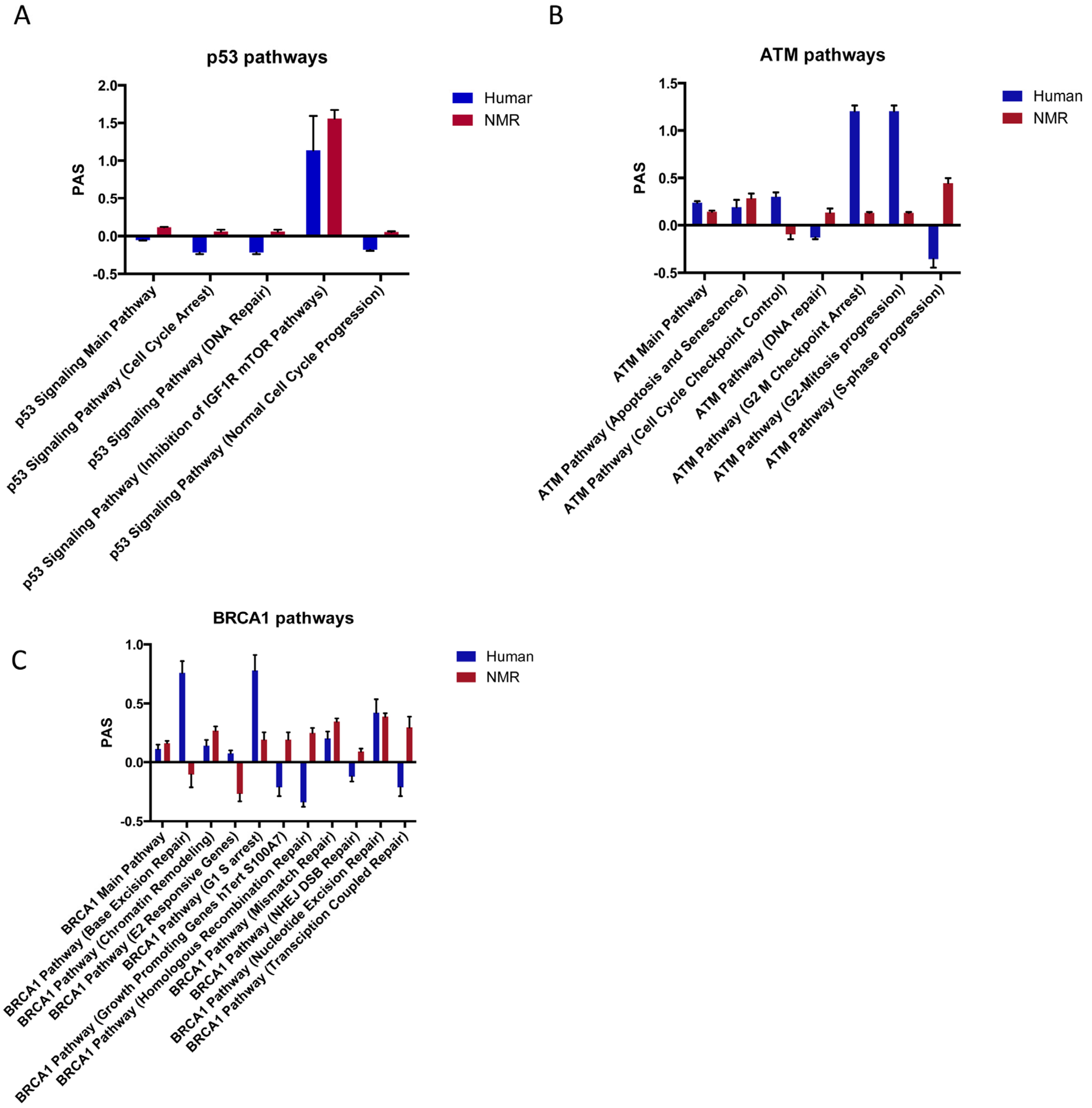

Figure 1S. Pathway activation strength (PAS) for signaling pathways. PAS values sub-pathways of (**A**) p53, (**B**) ATM, and (**C**) BRCA1, in human vs. mouse and NMR vs. mouse ($p < 0.05$). Positive PAS values reflect upregulated signaling pathways in human or NMR compared with mouse, and negative values represent down-regulated pathways. PAS scores of zero indicate similarly acting pathways in mouse compared with human or NMR. p53 Signaling Main Pathway p53 Signaling Pathway (Cell Cycle Arrest) p53 Signaling Pathway (DNA Repair) p53 Signaling Pathway (Inhibition of IGF1R mTOR Pathways) p53 Signaling Pathway (Normal Cell Cycle Progression) -0.5 0.0 0.5 1.0 1.5 2.0 p53 pathways PAS.

Supplementary Tables

Please browse the links in Full Text version of this manuscript to see Supplementary Tables 1-9. An Excel spreadsheet containing 11 supplementary tables related to this study. The tables are in 11 tabs:

- Table S1. Alignment statistics for each RNA sequencing library
- Table S2. Genes encoding proteins involved in DNA repair
- Table S3. Essential DNA repair genes with higher expression in mouse than human and NMR
- Table S4. Essential DNA repair genes with higher expression in NMR than mouse
- Table S5. Essential DNA repair genes with higher expression in human than mouse
- Table S6. Pathway activation scores for signaling pathways human vs. mouse
- Table S7. Pathway activation scores for signaling pathways NMR vs. mouse
- Table S8. Enriched GO biological process terms with higher expression in human than mouse
- Table S9. Enriched GO biological process terms with higher expression in NMR than mouse
- Table S10. Enriched GO biological process terms with higher expression in mouse than human
- Table S11. Enriched GO biological process terms with higher expression in mouse than NMR

File format: XLSX size 92 KB.

Availability of supporting data

This project has been uploaded to GEO under the accession number GSE75606. Additional data files RNA-seq data from this manuscript were submitted to the Sequence Read Archive under accession code SRP053350 for the mice samples and SRP066912 for the human and NMR samples.
